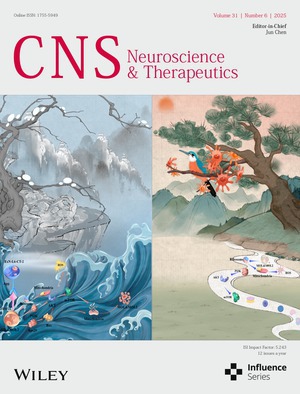# Front Cover

**DOI:** 10.1111/cns.70505

**Published:** 2025-06-29

## Abstract

The cover image is based on the article *Qishiwei Zhenzhu pills protect against cerebral ischemia via the P53/cytochrome C/apoptotic protease activating factor 1‐mediated mitochondrial apoptosis pathway* by Yinglian Song et al., https://doi.org/10.1111/cns.70476.